# Robust dose-response curve estimation applied to high content screening data analysis

**DOI:** 10.1186/s13029-014-0027-x

**Published:** 2014-12-10

**Authors:** Thuy Tuong Nguyen, Kyungmin Song, Yury Tsoy, Jin Yeop Kim, Yong-Jun Kwon, Myungjoo Kang, Michael Adsetts Edberg Hansen

**Affiliations:** Institut Pasteur Korea, Seongnam-si, Gyeonggi-do South Korea; University of California, Davis, USA; Samsung Medical Center, Seoul, South Korea; Videometer A/S, Horsholm, Denmark; Seoul National University, Seoul, South Korea

**Keywords:** High content screening, Dose response curve, Curve fitting, Sigmoidal function, Weighting function, Outlier detection

## Abstract

**Background and method:**

Successfully automated sigmoidal curve fitting is highly challenging when applied to large data sets. In this paper, we describe a robust algorithm for fitting sigmoid dose-response curves by estimating four parameters (floor, window, shift, and slope), together with the detection of outliers. We propose two improvements over current methods for curve fitting. The first one is the detection of outliers which is performed during the initialization step with correspondent adjustments of the derivative and error estimation functions. The second aspect is the enhancement of the weighting quality of data points using mean calculation in Tukey’s biweight function.

**Results and conclusion:**

Automatic curve fitting of 19,236 dose-response experiments shows that our proposed method outperforms the current fitting methods provided by MATLAB®;’s nlinfit function and GraphPad’s Prism software.

## Introduction

In recent years, the need for automatic data analysis has been amplified by the use of high content screening (HCS) [1] techniques. HCS (also known as phenotypic screening) is a great tool to identify small molecules that alter the disease state of a cell based on measuring cellular phenotype. However, HCS always comes with an important caveat: there is little or no a priori information on the compound’s target. At the Institut Pasteur Korea (IP-K), we have applied high content screening using small interfering RNA (siRNA) [2,3] at the genome scale. More recently, we applied the siRNA knockdown strategy for each gene from the genome and studied their effect on a given molecule producing a clear response on a cellular assay, to look for synergestic effects of a drug and target. Performing large-scale curve fitting for the biological activity of a large compound library can lead to a great number of dose-response curves (DRCs) that require proper fitting. The overall process of fitting, rejecting outliers, and data point weighting of thousands to millions of curves can be a significant challenge. The quality of a dose-response curve fitting algorithm is a key element that should help in reducing false positive hits and increasing real compound hits.

Several solutions [4-9] have been proposed to perform curve fitting. One of the widely used nonlinear curve fitting algorithms was introduced by Levenberg-Marquardt (LM) [5,6]. This method belongs to the gradient-descent family. However, due to the sensitivity of the method, the data quality and the initial guess, the curve fitting algorithm is unfortunately susceptible to becoming trapped in local minimums. To obtain proper curve fitting results, there is a need for automatic outlier handling, usage of predefined initial curves, and adaptive weighting for data points [10,11]. All of these demands can, without doubt, be applicable to large sets of data. Nonlinear and non-iterative least square regression analysis was presented in [7] for robust logistic curve fitting with detection of possible outliers. This non-iterative algorithm was implemented in a microcomputer and assessed using different biological and medical data. A review of popular fitting models using linear and nonlinear regression is given in [4]. The study serves as a practical guide to help researchers who are not statisticians understand statistical tools more clearly, especially dose-response curve fitting using nonlinear regression. This book also describes the robust fitting algorithm and the outlier detection mechanism used in the commercial software GraphPad Prism®;. In [8], an automatic best-of-fit estimation procedure is introduced based on the Akaike information criterion. This best-of-fit model guarantees not only the estimation of the parameters with smallest sum-of-squares errors but also good prediction of the model.

Simulated data for DRC estimation was used in [9] to examine the performance of the proposed Grid algorithm. The peculiarity of the Grid algorithm is that it visits all the points in a grid of four curve parameters and searches for the point with the optimum sum-of-squares error. A coarse-to-fine grid model and a threshold-based outlier detection mechanism were used to make the algorithm more efficient. This paper also provided Java-based software together with a sample dataset for academic use. However, the software is applicable only when the measurements are taken without replicates (one data point at each concentration). Furthermore, various popular computer software and code packages have been presented for DRC fitting such as the DRC package in R [12], the nlinfit function in MATLAB®; [13], and the XLfit add-in for Microsoft®; Excel®; [14].

In this paper, robust fitting and automatic outlier detection based on Tukey’s biweight function are introduced. This method was developed to automate nonlinear fitting of thousands of DRCs performed in replicates, detect the outliers automatically, and initialize the fitting curves robustly.

## Background and method

### Background

**Figure 1:**
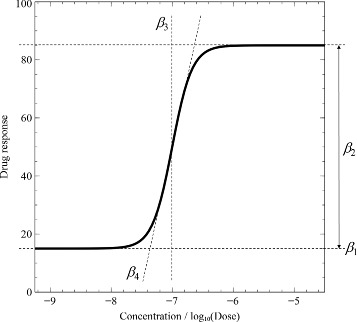
**A four-parameter dose-response curve.** *β*_1_,*β*_2_,*β*_3_, and *β*_4_ are the floor, the window, the shift, and the slope, respectively.

In drug discovery, analysis of the dose-response curve (DRC) is one of the most important tools for evaluating the effect of a drug on a disease. The DRC can be used to plot the results of many types of assays; its *X*-axis corresponds to the concentrations of a drug (in *log* scale), and its *Y*-axis corresponds to the drug responses. The function of DRC can be varied with different number of parameters, but the four parameter model is the most common:

(1) $$  f(x,\boldsymbol{\beta}) = \beta_{1} + \frac{\beta_{2}}{1 + \exp{\left(-\frac{\beta_{3} - x}{\beta_{4}}\right)}},  $$

where *x* is the dose or concentration of a data point; ***β*** represents the four parameters *β*_1_, *β*_2_, *β*_3_, and *β*_4_; *β*_1_ is the floor - the efficacy - which shows the biological activity without a chemical compound; *β*_2_ is the window - the efficacy - which shows the maximum saturated activity at high concentration; *β*_3_ is the shift - the potency - of the DRC; and *β*_4_ is the slope - the kinetics. Figure 1 shows an illustration of a response curve and its four parameters.

### Basic computation of curve fitting

The goal of a curve fitting algorithm is to solve a statistically optimized model that best fits the data set. Because the DRC function is nonlinear, an iterative method is considered to optimize parameters. In this section, a basic viewpoint is presented to approach the proposed ideas in our method. First, let *χ* be the function of the fitting parameter ***β*** which will be determined via function minimization:

(2) $$  \chi(\boldsymbol{\beta}) = {\sum_{i}^{N}} \rho\left(\frac{y_{i} - f(x_{i}, \boldsymbol{\beta})}{\sigma_{i}} \right),  $$

where *ρ*(*z*) is an error estimation function of a single variable *z*; *σ* is a normalization value; and *N* is the number of data points. To minimize (2), the Newton method is used to search for zero crossing of the gradient:

(3) $$  \boldsymbol{\beta}_{t+1} = \boldsymbol{\beta}_{t} + D^{-1}[-\nabla\chi(\boldsymbol{\beta}_{t})].  $$

Hence, we need to find the gradient and the Hessian matrix *D* of *χ*. The gradient of *χ* with respect to ***β***={*β*_1_,*β*_2_,*β*_3_,*β*_4_} is computed as

(4) $$  \frac{\partial\chi}{\partial\beta_{k}} = - {\sum_{i}^{N}} \frac{1}{\sigma_{i}} \psi(z) \frac{\partial f(x_{i}, \boldsymbol{\beta})}{\partial\beta_{k}},  $$

where *ψ*(*z*) is the derivative function of *ρ*(*z*) with *z*=(*y*_*i*_−*f*(*x*_*i*_,***β***))/*σ*_*i*_. The Hessian matrix is calculated using

(5) $$ {\small{  \frac{\partial^{2}\chi}{\partial\beta_{k} \partial\beta_{l}} = {\sum_{i}^{N}} \frac{1}{{\sigma_{i}^{2}}} \psi'(z) \frac{\partial f(x_{i}, \boldsymbol{\beta})}{\partial\beta_{k}} \frac{\partial f(x_{i}, \boldsymbol{\beta})}{\partial\beta_{l}} - \frac{1}{\sigma_{i}} \psi(z) \frac{\partial^{2} f(x_{i}, \boldsymbol{\beta})}{\partial\beta_{k} \partial\beta_{l}},}}  $$

where the second derivative term is negligible compared to the first derivative. Let

(6) $$  a_{kl} = \frac{\partial^{2}\chi}{\partial\beta_{k} \partial\beta_{l}}~\text{and}~b_{k} = \frac{\partial\chi}{\partial\beta_{k}},  $$

where *a*_*kl*_ and *b*_*k*_ are elements of matrices *A* and *B*, respectively; then instead of directly inverting the Hessian matrix, (3) can be rewritten as a set of linear equations:

(7) $$  \sum_{l=1}^{4} a_{kl}\delta\beta_{l} = b_{k},  $$

where *δβ*_*l*_ is changed at every iteration. Afterwards, to solve our fitting problem, we define

(8) $$  \rho(z) = \frac{1}{2} z^{2}~\text{and}~\psi(z) = z  $$

and obtain *a*_*kl*_ and *b*_*k*_ using least squares. The Levenberg-Marquardt (LM) method [5,6,15] solves (7) by defining a positive value *λ* to control the diagonal of the matrix *A*:

(9) $$  a'_{kk} = a_{kk}(1 + \lambda)~\text{and}~a'_{kl} = a_{kl}\;(k \neq l).  $$

The iteration steps of the LM method can be summarized as follows:

1. Evaluate *χ*(***β***) and define a modest value for *λ*, i.e., *λ*=0.001;
2. Solve (7) with *A* substituted by *A*^′^ in (9), and evaluate *χ*(***β***+*δ****β***);
3. If $\chi (\boldsymbol {\beta } + \delta \boldsymbol {\beta }) \geqslant \chi (\boldsymbol {\beta })$, increase *λ* by a factor of 10; else, decrease *λ* by a factor of 10 and update ***β***←***β***+*δ****β***;
4. Repeat steps 2 and 3 until *χ*(***β***) converges, and the return ***β***.

### Outlier detection

**Figure 2:**
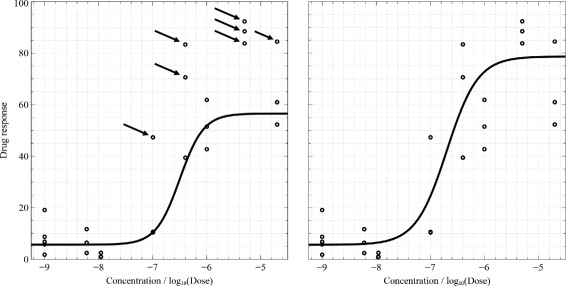
**Influence of noise (the arrows show the outliers).** Fitting on the left assigns low weights to the outliers to disregard them. Fitting on the right considers the outliers as useful data points and gives higher weights to these points.

In most cases, it is difficult to estimate the parameters, either due to noise in the observations or because the experimental design might give rise to ambiguities in the parameters of the DRC. There is a need for an outlier detection mechanism to cope with noise before fitting curves. Figure 2 shows the effect of outliers in the data. There are eight different concentrations, five replicates at the first concentration, and three replicates at the remaining concentrations. It is likely to become noisy when the number of data points increases. Seven outliers (the solid arrows show these outliers in the figure) can change the fit of the curve dramatically. These seven points in the left figure have lower weights (outliers detected) than those in the right figure.

In our problem, we initialize the fitting parameters ***β***_*o*_ (disregarding outliers) by finding the best fitted curve using

(10) $$  \rho(z) = |z|~\text{and}~\psi(z) = sign(z)  $$

instead of (8) which is used without outlier detection. Herein, we proposed (10) to reduce the impact of outliers on the fitting results by considering absolute errors and sign-only derivatives. The key difference between (10) and (8) is that the derivative function: *ψ*(*z*)=*sign*(*z*) results in −1 (negative) or 1 (positive), which controls the gradient in the direction of having a higher number of negatives/positives, whereas *ψ*(*z*)=*z* judges the gradient based on the distance between the estimated and actual values. Therefore, (10) is able to disregard the points having a lower number of negatives/positives (see Figure 2, on the left), and (8) considers the points at far distances although these points are given low impact (see Figure 2, on the right). Levenberg-Marquardt and other conventional nonlinear curve fitting algorithms are based on derivative calculation, and the quality of their solutions notably depends on data quality (i.e., outliers) and the initial guess. To have good fitting, outliers and the initial guess have to be manually detected and defined. Accordingly, these conventional algorithms are very difficult to automate and be made to yield good solutions in thousands of DRCs. Based on (10), outliers can be effectively weighted and a robust initial guess is automatically determined at the beginning of the fitting process. The method of weighting data points is described in the next section.

### Curve fitting with weighting function

Because all fitting parameters of the DRC are very important in understanding and assessing the effect of a chemical compound, it is essential to have a method that can estimate the curve in a robust way, i.e., coping with outliers and assigning weights to data points. Initially all data points are supposed to have equal weights. However, this idea does not hold in many practical occasions. Therefore, a least squares method tends to give unequal weighting to data points, e.g., points that are closer to the fitted curve would have higher weighting values. In standard weighting, minimizing the fitting error (sum-of-squares) of the absolute vertical distances is not appropriate: points having high response values tend to have large deviations from the curve and so they contribute more to the sum-of-squares value. This weighting makes sense when the scattering of data is Gaussian and the standard deviation among replicates is approximately the same at each concentration.

To overcome the situation in which data spreads differently at concentrations, various weighting techniques are considered, including relative weighting, Poisson weighting, and observed variability-based weighting [4]. Relative weighting extends the idea of standard weighting by dividing the squared distance by the square of the corresponding response value *Y*; hence, the relative variability is consistent. Similarly, Poisson weighting and weighting by observed variability use different forms of dividing the response value *Y*. Indeed, minimizing the sum-of-squares might yield the best possible curve when all variations obey a Gaussian distribution (without considering how different the standard deviations at concentrations are). However, it is common for one data point to be far from the rest (caused by experimental mistakes); then, this point does not belong to the same Gaussian distribution as the remaining points and it contributes erroneous impact to the fitting. The Tukey biweight function [10] was introduced to reduce the effect of outliers. This weighting function considers large residuals and treats them with low weights, or even zero weights, so that they do not sway the fitting much. In this section, we present a modification of the Tukey function and apply it to our fitting.

Let *ω*(*r*) be the weight of a data point that has a distance to the curve (residual) of *r*; then, the biweight function is defined as

(11) $$ \omega(r) =\left\{ \begin{aligned} &\left[ 1 - \left(\frac{r}{c} \right)^{2} \right]^{2}, \quad |r| < c \\ &0, \qquad\qquad\qquad\, |r| > c \end{aligned}\right.  $$

where $c = 6 \times median\left (\{r_{i}\}_{i=1}^{N} \right)$, 6 is a constant defined by Tukey, and *N* is the number of data points. This function totally ignores or gives zero weighting to the points having residuals larger than six times the median residual. Nevertheless, when the experimental data contains a great deal of noise (which usually occurs in biological and medical assays), it can fall on a normal distribution easier than when they contain little noise. When our data approaches a normal distribution, the mean of residuals is a better choice than the median of residuals (the median is useful if the data has extreme scores). In our case, based on experimental situations, we decided to use $c = 6 \times mean\left (\{r_{i}\}_{i=1}^{N} \right) = 6\bar {r}$. Figure 3 shows the fitting results of using the *median* and *mean*. The sum-of-squares error produced by using the *mean* is significantly better than by using the *median*. Additionally, the curve fit by using the *mean* looks more satisfactory than by using the *median*. Hence, the curve fitting algorithm with the modified Tukey biweight function can be summarized as follows:

1. Determine the distance from each data point to the curve, called the residual, *r*;

**Figure 3:**
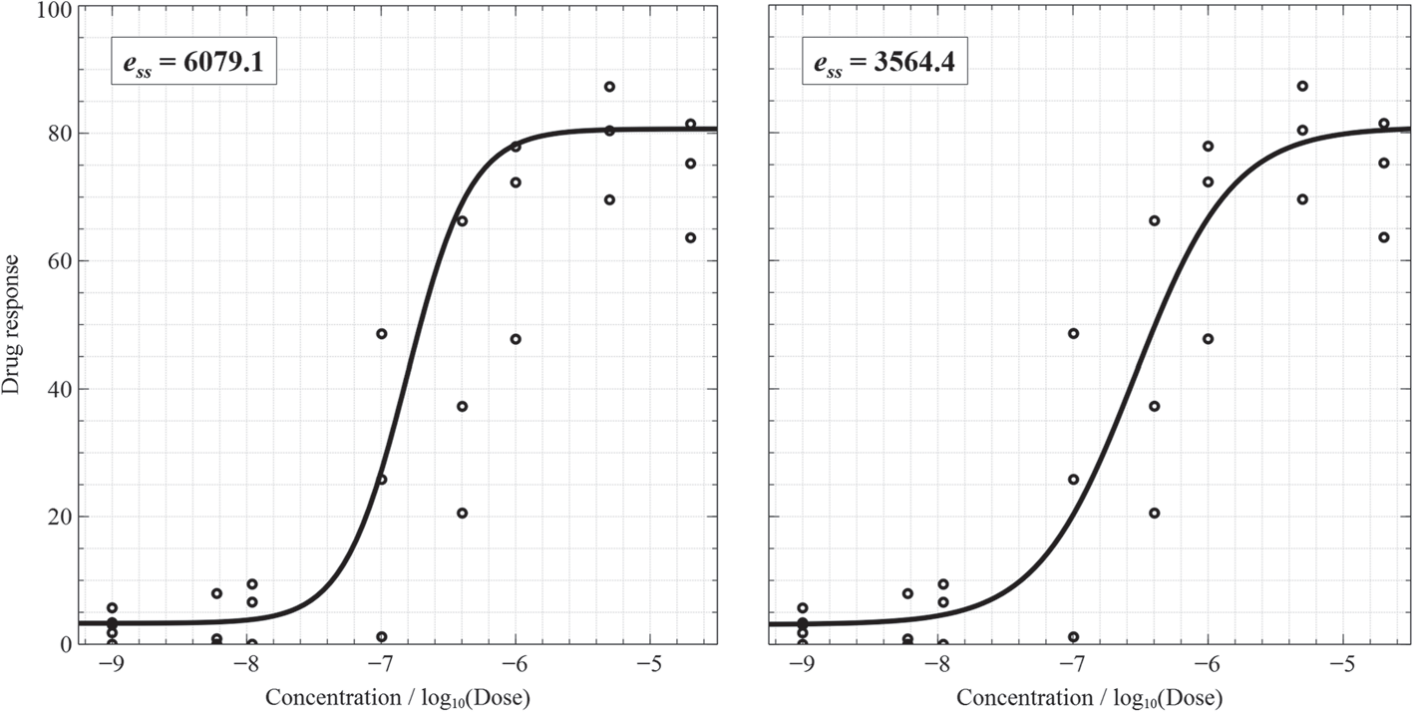
**Results of using the median (left) and mean (right) calculations in the Tukey biweight function.**
2. Calculate the weight of each point using (11) with $\bar {r}$ applied;
3. Assign new values to data points based on their weights.

In addition, other robust weighting functions can be considered such as Andrews, bisquare, Cauchy, Fair, Huber, logistic, Talwar, and Welsch [16]; however, the Tukey biweight function is known for its reliability, and it is generally recommended for robust optimization [17]. As we show further, the use of the Tukey biweight allows for improved DRC fitting results (see the Results section).

### Robust univariate DRC estimation algorithm

The proposed algorithm is conceptually easy to implement and robust to outliers. Combining ideas from the previous sections, the algorithm consists of the following steps:

1. Find the initial curve with outlier detection by executing the LM algorithm and applying (10) instead of (8);
2. Based on the curve obtained, calculate the Tukey biweights of data points using (11);
3. Based on the obtained weights, execute the LM algorithm in the basic computation section and consider the weights of the data points.
4. Repeat steps 2 and 3 for a predefined number of iterations or until convergence.

## Results

We have previously shown the ability to use the high content genome-wide silencing RNA (siRNA) screening approach on various cellular models [2,3]. The recent combination of drugs (at different concentrations) and siRNA (approximately 20,000) lead us to look for an automatic method to characterize a large number of dose-response curves obtained in those experimental conditions. Using MATLAB®;, a curve fitting algorithm was implemented. We compared the fitting performance of our method to that of the nlinfit function in MATLAB®; 2013a and the robust DRC fitting in GraphPad Prism®; 6.0.

In our experiments, we used eight different concentrations 0, 0.005, 0.01, 0.1, 0.4, 1, 5, and 20 *μM* with five replicates at concentration 0 and three replicates at the other concentrations (26 data points in total). In this manuscript, the concentrations were plotted over the *X*-axis in *log* unit. The *Y*-axis shows the drug response, which was normalized in the range of 0 to 100. The initial values of the parameters floor, window, shift, and slope used in the MATLAB®; nlinfit function were defined based on values of data points as *min*(*Y*), *max*(*Y*)−*min*(*Y*), *average*(*X*), and –0.6, respectively. By default, the algorithm uses bisquare (also known as Tukey biweight) as the robust weighting function. Indeed, MATLAB®; applied the Levenberg-Marquardt (LM) algorithm and iterative reweighted least squares [16] for robust estimation. Accordingly, the nlinfit represents the case of using the traditional LM algorithm and Tukey biweight function where (8) and the median function are utilized. For our algorithm, we defined the initial DRC parameters in the first step (outlier detection) as in nlinfit, and then those parameters were corrected using the outlier detection step and used for the consequent fitting steps. According to step 4 of our DRC estimation algorithm, the number of iterations was predefined as 50, and the error tolerance for convergence was 0.0001.

**Figure 4:**
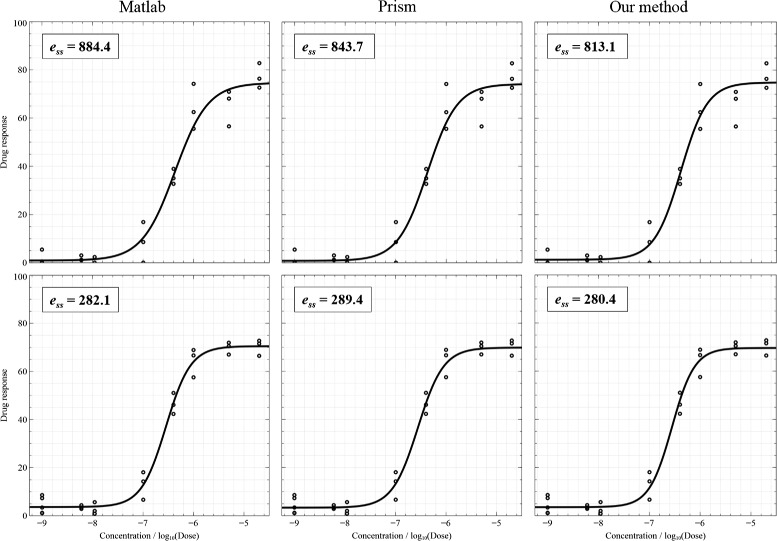
**Two results of no outliers and good fitting.**

**Figure 5:**
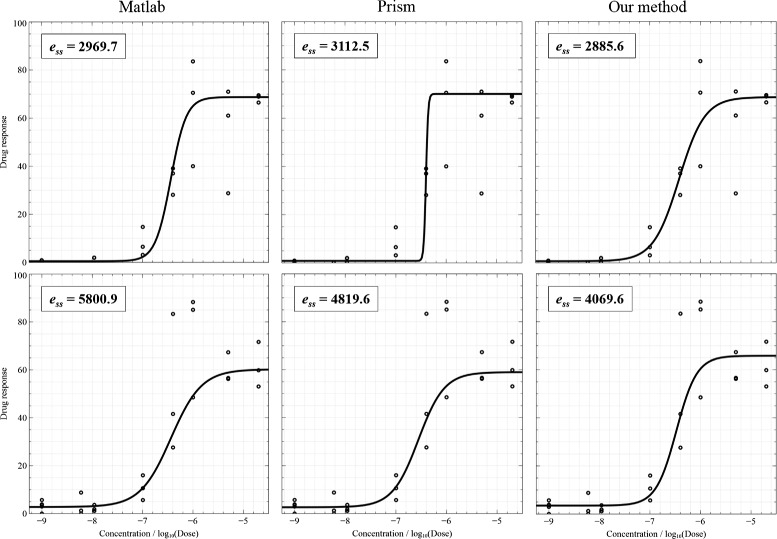
**Two results of outliers.**

**Figure 6:**
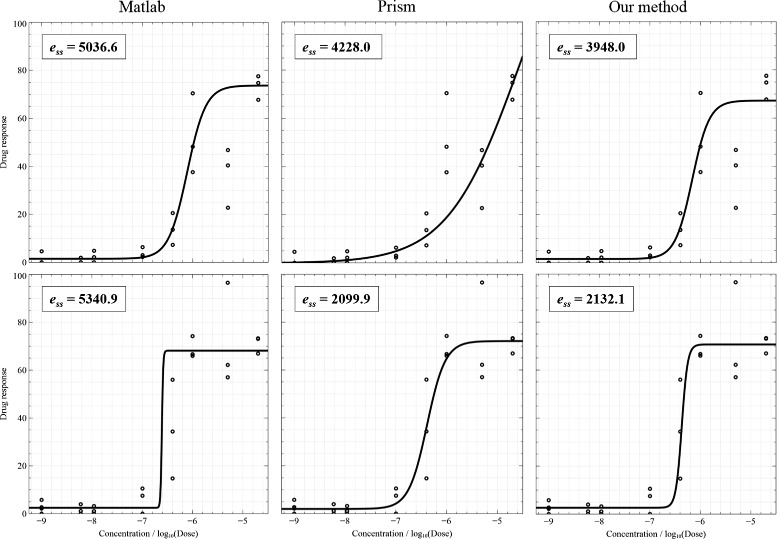
**Two results of outliers and bad fitting.**

Figure 4 shows two examples of fitting when data points do not include outliers and the results of three fitting algorithms are acceptable. Curve fitting results were presented together with sum-of-squares errors (on the top-left corner) which are calculated by taking the sum-of-squares of differences between the actual *Y* and the estimated *Y*. Smaller errors indicate better results. The plotting results are shown from left to right: MATLAB®; nlinfit, Prism®; robust fitting, and our method, respectively. Figure 5 illustrates the cases of the presence of outliers. For points inside the interval from −6.5 to −5 (*log* unit), the variation of measurements is high. In this figure, the first result of MATLAB®; nlinfit demonstrate an ambiguity of the shift parameter: the *log* of IC50 should be shifted to the right to cross the mean point in the middle of the plot. The first plot of Prism®; presents a poor DRC due to the high steep slope. Figure 6 displays the cases where outliers appear and lead to bad fitting. Prism®; was completely unable to fit the first curve, but our method handled the data points very well. Additionally, the second plot of MATLAB®; nlinfit shows an ambiguity of the shift parameter and a high steep slope.

In drug discovery and genome-wide data analysis, curve parameters, especially the shift, act as a crucial factor in determining the target candidates. Therefore, poor outcomes of the DRC fitting algorithm might greatly affect the analysis of the whole genome, which leads to difficulty finding the targets. To evaluate the performance of different DRC fitting algorithms on a large-scale, we assessed 19,236 curves which were obtained from five microarray slides. Table 1 shows fitting errors for the five slides with the corresponding number of curves 3885, 3887, 3865, 3875, and 3724 from each slide. Averages and standard deviations of the normalized sum-of-squares errors (the normalized error is calculated by dividing the actual error by the maximum one) were also included in this table with the comparison of the four methods. Herein, in addition to comparing our method (using mean calculation in the Tukey biweight function) to MATLAB®; and Prism®;, we also compared to our method, which uses the median for calculation of (11) to prove that the modified Tukey biweight function can significantly improve the fitting. Our method mostly yielded the best average errors in all slides whereas MATLAB®; nlinfit was the worst. Prism®; gave better results than the method using median calculation.

**Table 1:** **Averages and standard deviations of the normalized sum-of-squares errors calculated based on the fitting results of 19,236 curves**

**Slide**	**Number of curves**	**Matlab**		**Prism**		**Ours (median)**		**Ours (mean)**	
		***μ***	***σ***	***μ***	***σ***	***μ***	***σ***	***μ***	***σ***
**1**	3885	0.920	0.134	**0.777**	0.213	0.855	0.210	**0.777**	0.217
**2**	3887	0.907	0.117	0.852	0.154	0.943	0.134	**0.817**	0.165
**3**	3865	0.916	0.112	0.844	0.161	0.922	0.155	**0.820**	0.170
**4**	3875	0.946	0.105	0.793	0.198	0.841	0.202	**0.777**	0.207
**5**	3724	0.908	0.144	**0.722**	0.234	0.820	0.246	0.755	0.239
**Average**		0.919	0.122	0.798	0.192	0.876	0.189	**0.789**	0.200

Boldface numbers indicate the best errors.

In summary, experimental comparisons show that our method (namely ‘Ours (mean)’ in the figure), which proposes automatic initialization of DRC parameters and modification of the Tukey biweight function (mean calculation), yields a satisfactory fitting of curves. It provides more accurate fitting than MATLAB®; nlinfit, where automatic initialization is not available and the default Tukey biweight function (median calculation) is used, by more than 14% in processing 19,236 curves. We also demonstrated that the method applying the automatic initialization and the default Tukey function (namely ‘Ours (median)’) did not yield results as good as those of ‘Ours (mean)’. Moreover, the better performance of ‘Ours (median)’ than that of MATLAB®; nlinfit implies that the automatic initialization of DRC parameters meaningfully improves the fitting process. Our method is superior to GraphPad Prism®; 6.0 by more than 1%. Although the error is slightly improved; we believe that, with the MATLAB®; implementation provided, our approach is easily automated and scalable to thousands of curves. It is able to process the entire genome data in less than two hours – approximately 360 milliseconds for a curve (with a computer configuration using an Intel Core i7 3.47 GHz CPU).

## Conclusion

We provide two improvements for the problem of DRC fitting: 1) increasing the accuracy of the initialization of DRC parameters with the use of outlier detection, and 2) improving the method of weighting for noisy data in the Tukey biweight function. Our method is adapted to the analysis of thousands of DRCs or more with the use of automatic outlier detection and initialization of curves. By experimentally comparing the results of our method to those calculated by the nlinfit function in MATLAB®; 2013a and the robust DRC fitting in GraphPad Prism®; 6.0, we found that the proposed approach yielded a superior estimation of curves to that of MATLAB®; and Prism®;.
